# Sexual and reproductive healthcare for women asylum seekers in Switzerland: a multi-method evaluation

**DOI:** 10.1186/s12913-018-3502-2

**Published:** 2018-09-14

**Authors:** Eva Cignacco, Friederike zu Sayn-Wittgenstein, Coline Sénac, Anja Hurni, Doris Wyssmüller, Jean Anthony Grand-Guillaume-Perrenoud, Anke Berger

**Affiliations:** 1 Department of Health Professions, Division of Midwifery, Bern University of Applied Sciences, Bern, Switzerland; 2 Faculty of Business Management and Social Sciences, University of Applied Sciences Osnabrück, Osnabrück, Germany; 3 Mamamundo Association, Bern, Switzerland

**Keywords:** Sexual and reproductive healthcare, Women asylum-seekers, Asylum Centre, Healthcare provision, Gender-sensitive care, Interpreter services

## Abstract

**Background:**

Forced migration significantly endangers health. Women face numerous health risks, including sexual violence, lack of contraception, sexually transmitted disease, and adverse perinatal outcomes. Therefore, sexual and reproductive healthcare is a significant aspect of women asylum seekers’ health.

Even when healthcare costs of asylum seekers are covered by the government, there may be strong barriers to healthcare access and specific needs may be addressed inadequately. The study’s objectives were a) to assess the accommodation and healthcare services provided to women asylum seekers in standard and specialised health care, b) to assess the organisation of healthcare provision and how it addresses the sexual and reproductive healthcare needs of women asylum seekers.

**Methods:**

The study utilised a multi-method approach, comprising a less-dominant quantitative component and dominant qualitative component. The quantitative component assessed accommodation conditions for women in eight asylum centres using a survey. The qualitative component assessed healthcare provision on-site, using semi-structured interviews with health and social care professionals (*n* = 9). Asylum centres were selected to cover a wide range of characteristics. Interview analysis was guided by thematic analysis.

**Results:**

The accommodation in the asylum centres provided gender-separate rooms and sanitary infrastructure. Two models of healthcare were identified, which differed in the services they provided and in their organisation: 1) a standard healthcare model characterised by a lack of coordination between healthcare providers, unavailability of essential services such as interpreters, and fragmented healthcare, and 2) a specialised healthcare model specifically tailored to the needs of asylum-seekers. Its organisation is characterised by a network of closely collaborating health professionals. It provided essential services not present in the standard model. We recommend the specialised healthcare model as a guideline for best practise.

**Conclusions:**

The standard, non-specialised healthcare model used in some regions in Switzerland does not fully meet the healthcare needs of women asylum seekers. Specialised healthcare services used in other regions, which include translation services as well as gender and culturally sensitive care, are better suited to address these needs. More widespread use of this model would contribute significantly toward protecting the sexual and reproductive integrity and health of women asylum seekers.

**Electronic supplementary material:**

The online version of this article (10.1186/s12913-018-3502-2) contains supplementary material, which is available to authorized users.

## Background

At the end of 2016, about 65.6 million people around the world were forcibly displaced, of which 2.8 million were being processed for asylum [1]. Forced migration significantly endangers the health of fleeing people [2]. Mass accommodation in camps and inadequate hygiene can lead to the transmission of diseases [3–5], including sexually transmitted diseases and HIV/AIDS [6]. Sexual and reproductive health (SRH) in the context of forced migration is, however, often not the focus of attention of healthcare providers and policy makers [7–9]. Health risks for women are particularly high, as indicated by the high mortality of female refugees [10]. Among fleeing women, the risk of being exposed to gender-based violence during their migration journey is considerable [1, 11]. A high proportion of migrant women are of reproductive age [12] and suffer from lack of contraception, unsafe abortion, and lack of access to pregnancy and postpartum healthcare [13].

Migrant women also have a higher risk of adverse perinatal outcomes, including intrauterine foetal death [14], stillbirths [13], premature births, and aborted pregnancies [15, 16]. Furthermore, their neonates are at higher risk for admission to intensive care treatment [17]. The multitude and specific types of severe problems show that SRH is a significant aspect of the health of women asylum seekers and must be taken into consideration in their healthcare in the country of arrival [7, 13].

The process of how refugees are received in the hosting country and the living standards afforded to them vary widely [18]. In Switzerland, the asylum process is characterized by two stages. After arrival, asylum seekers enter first-access centres, where it is determined whether Switzerland or another European country is responsible for their asylum process, based on the Dublin Regulation [19]. After 90 days they are transferred to a follow-up asylum centre [20], where they either wait for the decision on their asylum application or are transferred to an individual lodging in a pre-determined community until a decision is made. The costs of health insurance for asylum seekers are covered by cantonal governmental bodies. In comparison to Germany [21], access to standard healthcare is provided free to asylum seekers and is as readily available as it is for Swiss residents. However, in the context of the asylum-seeking process, questions of ease of access of healthcare service and its actual use [22] arise. Healthcare might not address the specific healthcare needs of asylum seekers [23] traumatised by sexual violence [24]. In addition, women asylum seekers may be judged on the basis of negative cultural gender stereotypes related to women and problems specific to female refugees [25]. These issues and the higher health risks of women asylum seekers create a demand for specific SRHC and for accommodation facilities in hosting countries [26]. Specialised healthcare services for female migrants may be beneficial in reducing barriers to healthcare [23], as these programs are designed to actively approach women asylum seekers in a culturally sensitive manner and usually include translation services [27].

In order to understand how migrant women might access SRHC, by whom they are cared for and if their specific SRHC needs are addressed adequately, our study objectives were: a) to assess the accommodation and healthcare services provided to women asylum seekers in standard and specialised health care, b) to assess the organization of healthcare provision and how it addresses the sexual and reproductive healthcare needs of women asylum seekers.

## Methods

### Design

The study utilised a multi-method approach, comprising a less-dominant quantitative component and dominant qualitative component. The quantitative component assessed the accommodation conditions for women in eight asylum centres by means of a survey. The qualitative component assessed healthcare provision on-site, using semi-structured interviews with centre staff members. The qualitative interviews provided contextualized information on healthcare provision on-site, the perceived needs of women asylum seekers, and perceived problems in the provision of healthcare.

### Selection of asylum centres

We considered centres for inclusion in the study if they accommodate families, single women, and infants. Emphasis was placed on selecting asylum centres which cover the broad range of attributes that can be found in Switzerland, so as to allow for comparisons. These included: a) first-access centres, which provide accommodation for only 30 days, and follow-up access centres, which provide accommodation up to one year, b) three Swiss cantons representing all Swiss language regions (German, French, Italian), and c) urban and rural centres administered by federal or local (cantonal) governments. Out of a total of 72 centres in Switzerland we selected a purposive sample of eight, which varied in these attributes.

### Setting

The interviews took place within the facilities of the purposively selected eight centres. Of these eight selected, two were first-access centres, located in the Italian-speaking part of Switzerland, directly next to the border to Italy (Ticino, T1, T2; Table 1). These centres mainly host asylum seekers from the Middle East and Central Asia (including Iraq, Afghanistan, Pakistan) and sub-Saharan Africa, who crossed the Mediterranean Sea to reach Italy and then continued on to Switzerland. Six of the total eight selected centres were follow-up access centres. The six follow-up centres can be broken down into four centres located in the German-speaking canton of Bern (B1-B4) and two in the French-speaking canton of Vaud (V1, V2). Six of the total eight centres were administered by the cantonal government and two by the federal government. Three centres were located in urban areas and five in rural areas (Table 1).

**Table 1:** Characteristics of centres for asylum seekers in three Swiss cantons

	Bern				Vaud		Ticino	
	B1	B2	B3	B4	V1	V2	T1	T2
First access or follow-up centre	follow-up	follow-up	follow-up	follow-up	follow-up	follow-up	first-access	first-access
Government responsible	local	local	local	local	local	local	federal	federal
Setting	rural	rural	rural	rural	urban	urban	urban	rural
Capacity (residents)	100	180	140	80	165	92	134	50
Current residents (May 2017)								
Male	60	107	86	48	no data	56	67	11
Female	25	27	39	15	no data	22	12	15
Number of carers								
Male	10	6	3	8	2	2	11	3
Female	3	2	4	4	7	1	10	5
Professions and number of informants	child healthcare nurse (1)	medical practise assistant (1)	nurse (1)	paediatric nurse (1)	nursing and midwifery (1)	nurse (1)	nurse (1)	paediatric nurse (1)
		nursing assistant SRC (1)		nursing assistant SRC (1)				
Security personnel present	No	No	Yes	No	Yes	Yes	Yes	Yes
24 hours/day	-	-	No	-	No	Yes	Yes	Yes
Number of bedrooms	23	58	28	23	79	40	16	3
Gender-separate (%)	11 (48%)	39 (67%)	11 (39%)	no data	0	28 (70%)	4 (25%)	0
Number of toilets	20	14	9	15	no data	10	6	2
Gender-separate (%)	8 (40%)	12 (86%)	9 (100%)	5 (33%)	0	0	4 (67%)	2 (100%)
Number of showers	18	11	9	12	no data	10	4	3
Gender-separate (%)	5 (28%)	11 (100%)	9 (100%)	5 (41%)	0	0	4 (100%)	2 (67%)
Number of rooms, gender-separate								
Male	no data	33	0	no data	0	0	no data	0
Female	no data	6	0	no data	0	0	no data	0
Floors, gender-separate	3	3	no data	no data	0	0	2	no data
Kitchens	16	7	2	1	11	5	1	1

*Note.* The eight centres were selected for maximal diversity and represent local (cantonal) and federal governance, first and follow-up access centres, as well as three Swiss cantons and language regions (German, French and Italian). Switzerland, 2017

### Development of survey and interview guides

To develop questions for the quantitative questionnaire (survey) and the interview guide we analysed relevant reports related to refugees and migrants in Switzerland. We considered research reports of the Swiss government [28, 29], various United Nations institutions [30–33], and Swiss academic institutions [34, 35]. We also drew on the expert knowledge of the research team.

The quantitative questions of the survey served to describe the infrastructure of the centres, which includes the number of rooms, sanitary facilities, floors, and whether they are gender-separate.

The qualitative interview guide contained questions on healthcare provision, i.e. about treatments and the collaboration with external healthcare providers. Questions were also developed to draw information on the experiences and perceptions of professionals caring for female asylum seekers. The qualitative interview guide was semi-structured and translated from German into French and Italian by two native speakers in the research team. We include the interview guide as Additional file 1.

### Selection of informants

All contacted administrators in charge of the eight selected centres agreed to an on-site visit of the primary investigator (EC) and two assistants (CS and DW), as well as to an interview on the occasion of the visit.

We interviewed healthcare professionals working in these centres. Their duties were manifold. The primary role of centre staff members was as caretakers. Additionally, they served as healthcare providers and looked after minor health issues. They are from varied professional backgrounds within the field of nursing or social work. One informant had a background in nursing as well as midwifery.

To further investigate particularities of healthcare for asylum-seeking persons in Canton Vaud, we conducted an additional interview with a specialist medical expert in the care of vulnerable patients (Centre des populations vulnerables, University Hospital Canton Vaud, Lausanne, Switzerland). All interviews were conducted individually. No third persons were present during the interviews.

### Interviewers

The principal investigator of the study (EC) and two assistants (CS and DW) carried out the nine interviews. All have professional backgrounds as midwives and all have both practical and research experience. All are female. EC is a professor of midwifery. CS and DW are practising midwives. The interviewers were not known to the informants prior to the interviews. The interviewers introduced themselves as midwives with a research mandate from the Swiss Federal Office of Public Health. Interviewers were assigned to language regions based on their native language, ensuring that interviews were carried out by native speakers in the respective languages, German, French, and Italian.

### Data collection

Ten days ahead of the visit we provided our contact persons from each centre with written information on the purpose of the study, the survey and interview guides, and an informed consent form. We collected the consent forms during the site visits, prior to the start of the interviews. Interviews took place between 1st April and 28th April 2017. The audio-taped interviews lasted 60–120 min. Field notes were made during the interviews to supplement the audio recordings.

### Data analysis

The data was coded by five coders (EC, FSW, AH, CS, and DW). To handle our multilingual data we defined the original and target languages and specified points of translation [36]. The data was transcribed in the original language in summarised form to allow for initial assessment and interpretation of the data during transcription [37]. German was the dominant language in the study and was used as the common language for analysis, developing codes and synthesising the data. The analysis was guided by thematic analysis based upon a realist epistemology [38]. We reviewed the transcripts as a team on three consecutive days to define central statements in the data. The statements in the data were used to derive codes and categories, which were subsequently integrated into suitable theory-driven themes, derived from literature and professional expertise. When indicated by the data, we summarised categories into appropriate higher-level themes. On these three days we discussed the codes extensively, until we came to an agreement which codes best describe the data and how they relate to each other. Field notes were used as a means of data source triangulation and provided additional support for our coding. Microsoft Visio was used to visualise the relationships within the data. We confirmed our data to be saturated when no new categories could be extracted [39].

## Results

### Quantitative findings

#### Accommodation, residents, and centre staff

The asylum centres in the study provided a total of 270 bedrooms of which 93 (34%) were gender-separate or assigned to individual families. Three centres also had gender-separate floors (Table 1, B1, B2, T1). Families generally shared one sleeping quarters. Other shared sleeping accommodations were gender-separate. Of the bathrooms, 53% of toilets and 54% of showers in the 8 centres were gender-separate. One centre did not have gender-separate toilets and showers, and for one centre no information was available (Table 1, V1, V2).

At the time of the interviews 739 asylum-seekers were living in the centres. Females made up 247 of the residents (33.4%). Of the females, 61.1% (*n* = 151) were of reproductive age (18 to 48 years of age), while 25.1% (*n* = 62) were children and adolescents (0 to 17 years of age) (Table 2). In the twelve-month period before data collection (April 2016 to April 2017), 56 children were born in these eight centres. The most common countries of origin of women asylum seekers were Eritrea, Ethiopia, Iraq, Afghanistan, and Syria.

**Table 2:** Relative frequencies of women asylum seekers by age group and civil status

	Proportion %
By age	
0-17	25
18-29	33
30-39	18
40-48	10
48+	14
By civil status	
Single	51
In partnership	3
Married, cohabiting	29
Married, separated/widowed	5
Divorced	8
Unknown	4

The number of employed centre staff members ranged from 3 to 21 per centre. Some centre staff members were health professionals. Of the eight centres, five had a security service. In three of these shelters security staff was present around the clock (Table 1).

### Qualitative findings

#### Characteristics and needs of women asylum seekers

In our interviews with centre staff members, we found that they see several commonalities among women asylum seekers with regard to their experiences, cultural background, and hopes. One of the main issues they noted was that the women often suffer from anxiety. They struggle with their insecure immigration status and are preoccupied with thoughts of family members left behind in their countries of origin. They require time to accommodate to their new surroundings and to build a trusting relationship with the centre staff members.

Although it was not systematically assessed by asking the women themselves, the staff members believe that some women were raped during their migration journey and suffer from problems associated with the rape, including unwanted pregnancy and sexually transmitted disease. This is particularly true for women from sub-Saharan Africa. However, these women usually do not volunteer information about their experience of being raped.

I think there are also women, they get pregnant because of violence, --rape, and don’t tell that to anybody. (Excerpt, Interview 3)

Their cultural background and having experienced rape or genital mutilation contributes to women wanting to be treated exclusively by a female gynaecologist.

Women don’t want to go to a male doctor and tell them what their problem is. (Excerpt, Interview 4)

Women who suffered genital mutilation are also observed to be reluctant to speak of their experience. Staff members often only recognise that a woman may have been circumcised when she asks for pain medication regularly every month during menstruation.

On the circumcision of women: We get too little information. It is still a subject that people do not talk about. (Excerpt, Interview 4)

Women who are circumcised very often have huge menstrual pains, and then we notice that they are circumcised because they ask for pain medication every month. (Excerpt, Interview 1)

Some centre staff members are highly aware that experiences of violence and genital mutilation require particular attention. However, they have no training in how to assess or refer potentially affected women to specialised settings. This lack of attention is perceived as a serious shortcoming.

Women refugees really get lost in the whole context of migration. We really have to pay special attention in our own actions that we do not lose sight of them. (Excerpt, Interview 1)

The asylum-seekers often do not speak a common language with the staff, such as English or French, which creates severe difficulties and limitations in communication.

Yes, we communicate by gesturing. Sometimes I even draw something or try to act out a scene. You have to be creative. (Excerpt, Interview 4)

#### Healthcare provision models for asylum seekers: Specialised versus standard care models

Two models of healthcare can be discerned. They differ in their organisation and the services they provide. We found a more basic set of services provided in the canton of Bern (standard healthcare model) and a more elaborate set of services provided in the cantons of Vaud and Ticino (specialised healthcare model), which are specifically aimed at addressing the needs of asylum seekers. The defining characteristic of the standard healthcare model found in Bern (B1-B4, Table 1) is that there is one primary care physician assigned to each individual centre, who is responsible for basic clinical assessment and for referring patients to other external healthcare providers, if needed. External healthcare includes obstetric and gynaecologic treatment. For treatments requiring services by several physicians the standard healthcare model has several shortcomings. It is unable to provide a frictionless continuity of healthcare and to ensure the exchange of pertinent patient information due to a lack of coordination between the parties involved. Certain staff members in asylum centres are also trained healthcare professionals, but mainly serve as caretakers and attend only to minor healthcare needs of residents, such as headaches or menstrual pains. They do not have the authority to decide on treatment or to receive and pass on patient information to other health professionals outside the centre. A further characteristic of the standard healthcare model is the lack of paid interpreter services for any kind of health consultations.

In contrast, in the specialised healthcare model found in Vaud (V1, V2, Table 1), asylum seekers are referred to an external healthcare centre that is dedicated exclusively to asylum seekers. These healthcare centres are administered by nurses who serve as primary care providers and case managers. They have the competence and autonomy to deliver primary healthcare and are charged with coordinating treatment with specialist physicians. The specialised healthcare centre provides interpreter services for all consultations, free of charge.

There is a kind of a gate-keeping nurse. So the model is really based on the primary care nurse. These are not quite nurse practitioners in the North American or Anglo-Saxon sense, because our nurses do not have the university title of Master of Science, Nurse Practitioners. Even so, they are the backbone of everything. (Excerpt, Interview 6)

The specialised healthcare centres are also integrated into a network of associated medical professionals, including primary care physicians, psychiatrists and paediatricians to whom the asylum seekers are referred by the nurses when required. Gynaecologists, however, who are essential for SRHC, are not part of the network of health professionals for women asylum seekers, as they are considered additional services that are not part of primary care. The exclusion of the most important healthcare professional for reproductive-age women is described as a shortcoming by the specialised healthcare nurses who were interviewed.

A specialised healthcare model can also be found in first-access centres in Ticino (T1, T2, Table 1), where nurses provide triage and basic healthcare. Nurses in these centres coordinate treatments with external healthcare providers, and there is a free exchange of pertinent patient information between the professionals involved in the care of the women.

#### Fragmented healthcare

The interviews with staff members in both healthcare models revealed that issues of fragmented healthcare may arise due to lack of information, lack of sensitivity of healthcare providers to the special needs of women asylum seekers, or due to required but unavailable services. These shortcomings are more pronounced in the model with standard healthcare services (B1-B4).

##### Inadequate information

In the first-access centres staff reported that asylum seekers, especially women, discard their medical documents and medications while in transit from Italy to Switzerland, out of fear of being registered as an asylum seeker in Italy.

Many of them tell us that they have thrown away the documents. They throw away all the documents and even the medication they got to hide everything. (Excerpt, Interview 8)

Many of the asylum seekers who arrive in the Italian part of Switzerland have already undergone medical examinations in Italy. However, due to the loss of this patient information, all tests have to be repeated in Switzerland.

Many women tell us they had ultrasound, they also did a blood test. But if you ask them if they can show you documents they have nothing! And that's a shame because the information is completely lost after crossing the border. (Excerpt, Interview 8)

The medical information is also not passed on by Italian authorities to the Swiss authorities.

We do not get any documents [from Italy], so we go through all the examinations and all the visits they've already done in Italy. (Excerpt, Interview 8)

Staff members also report that missing- or inadequate-information problems occur when asylum seekers transfer from first-access centres to follow-up centres. Patient information on general and reproductive health appears to be standardised, but the information passed on to the follow-up centres is sometimes found to be incomplete or unreadable.

It’s not standardised, what we receive. There are a few pages, but they get filled in very differently. And there are no standards. (Excerpt, Interview 1)

We receive medical documents in German, in Italian. My colleague and I do not speak Italian, so we must find a colleague who will translate. Often it is handwritten; it is often illegible. (Excerpt, Interview 5)

In first-access centres standardised information on pregnancy is obtained by using only one rudimentary questionnaire item “Pregnancy: Yes/No.” For pregnant asylum seekers who are transferred to the cantonal asylum centres, this minimal information is seen as a considerable risk factor.

There was a woman very advanced in her pregnancy who travelled from Basel to the asylum centre alone with her three children. That was a catastrophe. She could have had her child anywhere. We saw in her documents that on that day she had an appointment for a birth induction in Basel. We had no information about that. (Excerpt, Interview 1)

Issues with the availability of information are also found in the collaboration between follow-up centres and healthcare providers in the standard healthcare model. Because of physician-patient confidentiality, external healthcare providers do not share patient information with healthcare professionals in the asylum centres.

There is little exchange with the doctors. The problem is doctor-patient confidentiality. (Excerpt, Interview 4)

##### Lack of sensitivity to needs

Interviews indicated that there might be a lack of sensitivity to the special needs of women asylum seekers, as some staff members do not believe that women asylum seekers have special needs.

They are simply pregnant, and that is normal, and then you have a child, and that is normal, too. And whether you give birth in a birth centre or in a clinic doesn’t make a difference. The women simply want to have their child and are happy to receive medical support, but the women don’t need anything else. (Excerpt, Interview 4)

##### Unavailability of services

The unavailability of several essential services is another factor that may contribute to fragmented healthcare. In the canton of Bern, interpreter services are not funded by the government. Conducting a medical history, explaining medical treatments and ensuring patients’ medical compliance was found to be particularly challenging without interpreter services.

Certain medical and healthcare professionals are not part of regular healthcare provision or are difficult to get access to. Gynaecologists, for instance, are not part of the healthcare network for asylum seekers in the canton of Vaud (Table 3). Overburdened gynaecologists, among others, often refuse to accept asylum seekers as patients. Finding female gynaecologists or physicians also appears to be difficult. Services provided by midwives and community-based child healthcare nurses are available only on demand, but are not an integral part of standard care.

**Table 3:** Services available to asylum seekers in three Swiss cantons. Switzerland, 2017

	Bern	Vaud	Ticino
Interpreter	No	Yes	Yes
Shuttle bus for medical treatments	No	Yes	No
Gynaecologists	Yes	Yes (not standard)	Yes
Psychiatrists	Yes	Yes	Yes
Midwives	Yes (not standard)	Yes (not standard)	Yes (not standard)
Birth preparation courses	Yes (not standard)	Yes (not standard)	Yes (not standard)
Parenting coaches	Yes (not standard)	Yes (not standard)	Yes (not standard)
Family planning			
Condoms	Yes	Yes	Yes
Female contraceptives	Yes (at cost)	Yes (at cost)	Yes (at cost)
Abortions	Yes	Yes	Yes

When healthcare takes place outside of the asylum centres, the lack of free shuttle bus transport (Table 3) makes it difficult for pregnant asylum seekers to go to doctor’s appointments. Pregnant women need to find their way on their own to their doctor or be accompanied by centre staff members. Also, antenatal classes specifically tailored to migrant women are difficult to attend, as they are not offered close to the centres.

In regard to family planning, women have restricted access to contraceptives. Condoms are freely and easily available, but other methods of contraception have to be paid for partially or in full by the women asylum seekers. This restricted ability to use contraceptives makes women dependent on their partner. In contrast to the limited access to free female contraception, abortions are paid for by health insurance and can be accessed when needed.

## Discussion

Our finding that 61% of women asylum seekers and girls in the asylum centres under investigation were of reproductive age suggests SRHC as a vital element to be considered in the healthcare provision of this population. While we found adequate accommodation in most centres, some centres lacked gender-separate sanitary facilities. The special healthcare needs of this vulnerable group are addressed inadequately in the standard healthcare model, which does not include certain essential services, such as interpreter services.

### Accommodation and gender-appropriate support in asylum centres

Our finding that rooms and facilities are with few exceptions generally available in asylum centres in order to allow women to be separate from men indicates that accommodations for asylum seekers in Switzerland are in line with the recommendations of the European Parliament to take gender into account in order to improve security and safety for women and girl refugees [40]. The European Union Agency for Fundamental Rights [41] recommends separate accommodation and sanitary facilities to prevent gender-based violence. The gender separation within the centre may serve to prevent exposure to potential violent behaviour from men [42] and subsequent re-traumatisation.

However, gender-separate sanitary facilities and sleeping quarters are not available in all reception and transit centres in the European Union [40]. Our finding from Switzerland that seven out of eight centres investigated had gender-separate toilets and showers and that six out of eight centres had gender-separate sleeping quarters is encouraging. Ideally, gender-separate sanitary facilities and sleeping quarters should be available in all centres.

The employment of women staff members in all centres indicates that gender-appropriate support in terms of same-gender caretakers is ensured. The UNHCR [43] regards the availability of female staff in refugee camps as a precondition for the effective protection of women and girls. Lack of female staff is a significant barrier to ensuring sexual and reproductive health, as women refugees may forgo seeking medical care [44]. Similarly, women and girl refugees are less likely to report gender-based violence to men [44]. Having female staff members for women asylum seekers to confide in may make it easier to speak about potential rape experiences and other SRHC-related issues. This is particularly significant given the high risk of female refugees of being exposed to gender-based violence during flight [11, 33]. Our finding that female staff members are employed in all centres investigated is reassuring, as it increases the likelihood that SRHC-related matters are discussed and dealt with.

### Meeting SRHC needs of women asylum seekers: Standard versus specialised healthcare provision

The healthcare model with standard care as established in the German-speaking part of Switzerland profoundly impacted the provision of health services due to a) the unavailability of pertinent patient information, b) inadequate coordination between the asylum centre and external healthcare providers, and c) marginal use of midwifery and community-based child health services. These services are known for providing more individualized woman- and family-centred care [45]. The neglect of woman-centred care among women asylum seekers might be due to the phenomenon in industrialized countries that pregnant women of low socio-economic status are not comprehensively informed by their healthcare providers about having a choice in maternity care services, such as midwifery services or antenatal classes [45]. They therefore tend to comply with the prevailing medical or public clinical care model, which does not meet their special needs and can lead to worse perinatal outcomes [27, 45].

In the standard healthcare model physician-patient confidentiality was the main reason for external healthcare providers’ reticence to pass on patient information to asylum centres. Ensuring information exchange is essential to patient safety [46].

When healthcare is handled by multiple healthcare providers, models of managed care are required to ensure appropriate and timely treatment [47] and the availability of patient information [48]. In regard to the availability of patient information and managed care we found favourable conditions to be present when healthcare was specialised for the needs of asylum seekers, as we encountered in the model of the French- and Italian-speaking part of Switzerland, in which information was freely exchanged among professionals within the healthcare network. The coordination of treatment also facilitated a frictionless handoff of patients between healthcare providers. This ensures patient well-being and serves to give patients peace of mind and reassurance [48].

The study presented raised concerns that some professionals may not show appropriate sensitivity to the cultural, social, and psychological needs of women asylum seekers, as the informant seemed to suggest that in childbirth there are only medical needs that need to be attended to. This might be an indicator for a lack of cultural sensitivity. Cultural sensitivity among healthcare professionals is a concern frequently raised in regard to the healthcare of refugees [49–52]. It includes responsiveness to language barriers as well as to differential beliefs, perceptions and values in regard to health and illness which female asylum seekers may hold. A lack of cultural sensitivity among professionals has been linked to differences in health outcomes among minority groups [50].

Ensuring the availability and accessibility of essential healthcare-related services is crucial to continuity of healthcare. We noted the unavailability of certain essential services, of which we highlight three areas: a) interpreter services, b) female contraceptives and family planning, c) access to alternative healthcare professionals during pregnancy, such as midwifery services or antenatal classes.

Interpreters provide essential services to physicians, which, e.g., aids in obtaining patient histories [27, 53]. In our study we found that the unavailability of interpreter services in the standard healthcare model severely impacted the ability to conduct a clinical assessment. It also strongly curtailed the ability of healthcare professionals to explain medical treatments to asylum seekers and to ensure medical compliance. Not surprisingly, interpreter services are strongly recommended as part of refugee healthcare by numerous studies [54].

In regard to family planning we found that the main issue in the centres lies in the limited access to female contraceptives due to the costs involved in obtaining them. Asylum seekers were required to pay for female contraceptives partially or in full out of their allowance. Only condoms were available for free and easily accessible. Contraception that is mainly based on condom use is problematic, as it is contingent on the male partner’s approval and use. Given that the abortion rate among women asylum seekers in Switzerland is 2.5 times that of the Swiss population [15], the policy of not funding female contraceptives may need to be reviewed. Since funding for contraceptives was shown to increase the use of contraceptives among refugees in camps [55], it is probable that increased availability of female contraceptives will reduce the frequency of abortions. The reallocation of funding from free abortions toward free female contraceptives is likely to contribute to protecting asylum seekers’ health and to reducing abortions.

Non-physician healthcare professionals were not included as part of standard care in the asylum centres. The likelihood of various negative outcomes, such as foetal loss and episiotomy, is significantly reduced with midwife-led healthcare, while the likelihood of desirable outcomes, such as spontaneous vaginal birth, maternal breastfeeding, and maternal feelings of control are increased [56]. With its holistic view of healthcare and focus on health promotion [57], midwife care may be able to affect positive outcomes not only in the ante- and perinatal period, but also in child development after birth [58]. Similarly, midwife-led antenatal education can have a positive effect on psychological outcomes, such as a reduction in fear of childbirth and an increase in maternal self-efficacy [59], as well as physical outcomes, such as a reduction in caesarean sections and other interventions [60].

### Strengths and limitations

The present study is the first to investigate sexual and reproductive healthcare provision for female asylum seekers in asylum centres in Switzerland and, to our knowledge, in Europe.

We provide new insights on a subject that is of growing importance to nations around the world, which are receiving a growing number of refugees. Based on our investigation into the conditions of healthcare provision of a vulnerable population, we provide specific recommendations for the improvement of SRHC for female asylum seekers in asylum centres.

The study is limited by the approach to gathering data on women asylum seekers indirectly through interviewing centre staff members in asylum centres. A further limitation lies in the fact that qualitative inquiries are more strongly influenced by contextual factors and the subjective experiences of the informant than are quantitative studies. However, its exploratory nature allows for topics to be discussed that go beyond the initial interview questions. This facilitates the discovery of issues that are related to the phenomenon under study. Finally, data were collected from a purposive sample of asylum centres, which limits our ability to generalise the findings beyond asylum centres that feature the selected characteristics.

### Recommendations for healthcare practise for women asylum seekers

Based on our findings and discussion we suggest adaptations in the most important domains of asylum seekers’ healthcare: a) healthcare organisation, b) availability of services, c) continuing professional education. Our findings strongly suggest that specialised healthcare models are likely to be more effective in addressing the special health needs of a highly vulnerable patient population. They entail benefits to the free exchange of information, frictionless collaboration among healthcare providers and give asylum seekers the chance to make informed choices in treatment.

The integration of low-barrier access to specific support services may reduce the possibility of fragmented healthcare. Specifically, and most importantly, we recommend that funding should be provided for interpreter services to facilitate communication between healthcare providers and asylum seekers on complex healthcare topics. This will allow valuable patient information to be gained and ensure that patients understand and can adhere to their treatments. Finally, continuing professional postgraduate education that includes courses on culture and gender-sensitive care may aid in raising awareness of the circumstances and issues that women asylum seekers face.

While our recommendations seek to address the healthcare shortcomings we encountered in our research, their scope is limited to the healthcare context in Switzerland. It is evident that certain issues can only be addressed on a larger, EU-wide scale. Patient information exchange between Italy and Switzerland, for instance, was one of the problems that we discovered which requires cooperation across national borders. Several European directives and conventions concerning sexual and reproductive health have been proposed [61]. We recommend a closer collaboration and coordination of efforts between nations, as they are essential in ensuring that the policies, resources, and procedures are in place to support effective healthcare delivery for migrants.

## Conclusions

Asylum seeking women are a highly vulnerable population with specific healthcare needs that are often not met in standard healthcare provision. Specialised healthcare services, including translation services as well as gender and culturally sensitive care are crucial to protecting the sexual and reproductive integrity and health of women asylum seekers. A specialised healthcare model with integrated interpreter services would seem to address the SRHC needs of women asylum seekers more appropriately and reduce fragmentation of healthcare.

## Additional file

Additional file 1: Interview Guide. Translated interview guide for qualitative interviews with asylum centre staff. (DOCX 45 kb)

## Abbreviations

AB: Anke Berger
AH: Anja Hurni
CS: Coline Sénac
DW: Doris Wyssmüller
EC: Eva Cignacco
FSW: Friederike zu Sayn-Wittgenstein
JAG: Jean Anthony Grand-Guillaume-Perrenoud
SRH: Sexual and Reproductive Health
SRHC: Sexual and Reproductive Healthcare
