# Supplementary material for: Sexual and reproductive healthcare for women asylum seekers in Switzerland: a multi-method evaluation
Source: BMC Health Serv Res. 2018 Sep 14;18:712. doi: 10.1186/s12913-018-3502-2 (PMC6137714; doi:10.1186/s12913-018-3502-2)
Supplement: Supplementary file 1 — Interview Guide. Translated interview guide for qualitative interviews with asylum centre staff. (DOCX 45 kb) [file 12913_2018_3502_MOESM1_ESM.docx]

# Interview Guide

## Healthcare in the Asylum Centre

### Survey of the present health status:

- Is a general clinical assessment conducted for this target group? If yes, please indicate which kind (physical/psychological/gynaecological)?
- Is there a standardised assessment form?
- Is an interpreter consulted during assessment?

### Psychological healthcare:

- Is data on experiences with violence / trauma systematically collected?
- Is an interpreter consulted during this procedure?
- What is the course of action if a current traumatisation is found?

### Gynaecological healthcare:

- How can an asylum-seeking woman get information on contraception and family planning? How does she get information on consultations?
- Who can a woman go to if she has acute or chronic gynaecological problems (e.g. vaginal infections, bladder infections, menstrual problems, strong or foul-smelling discharge)?
- Where are prophylactic gynaecological examinations conducted?
- Is an interpreter consulted during the examination?

### Healthcare during pregnancy:

- How are the pregnancy check-ups organised?
- Is the woman familiarised with different models of healthcare during pregnancy? (e.g. freelance midwife or gynaecologist, hospital care)? Are pregnant women given a choice?
- Which options (e.g. internet access, information material, prenatal classes) do women have to inform themselves or exchange information about pregnancy?
- Whom can a pregnant woman contact for acute problems and emergencies?
- Which options does a pregnant woman with pregnancy issues (e.g. contractions or back pains) have, so she can be alone?

### Care during birth:

- How is the transport to the place of child birth organised (public transport, car)?
- Is the pregnant woman accompanied by someone? If yes, by whom? (Partner, friend, acquaintance, relative)

### Lying-in period:

- How is the community-based midwife care organised in the centre?
- Does a consultation take place with the aftercare midwife before birth?
- Does the woman in childbed live in a single room / room for families?
- Are there possibilities to get support for cooking / household chores?
- Does the woman in childbed receive follow-up support services (e.g. parent-child meeting)?

### Infant care:

- Are financial resources available for specific purchases (e.g. clothing, stroller, baby carrier towel)?
- How are appointments with the paediatrician organised in the first few weeks after birth?
- Whom can the mother turn to for acute problems (cry baby, illness)?

### Network:

With which other organisations or professionals is your centre in contact?

(Mark all that apply)

- Midwives □
- Gynaecologists □
- Paediatricians □
- Home healthcare service (Spitex) □
- Birth clinics, paediatric clinics, birth preparation helpers □
- Community-based child healthcare nurses □
- Social workers / social educationalists □
- Follow-up services (e.g. baby meetups, early development) □
- Intercultural interpreters □
- Authorities □
- Legal advisors □
- Other □

## Protection from Violence in the Asylum Centre

- Does your centre have a violence protection concept?
- How are women protected from gender-based violence and sexual assault/harassment?
- Whom can affected women turn to if they feel harassed? Are there any administrative hurdles?
